# Stress-testing development pathways under a changing climate: water-energy-food security in the lake Malawi-Shire river system

**DOI:** 10.1098/rsta.2021.0134

**Published:** 2022-04-18

**Authors:** Ajay G. Bhave, Declan Conway, Suraje Dessai, Andrew J. Dougill, David Mkwambisi

**Affiliations:** ^1^ School of Engineering, Newcastle University, Newcastle Upon Tyne NE1 7RU, UK; ^2^ School of Earth and Environment, Sustainability Research Institute, University of Leeds, Leeds LS2 9JT, UK; ^3^ Grantham Research Institute on Climate Change and the Environment, London School of Economics and Political Science, London WC2A 2AE, UK; ^4^ Malawi University of Science and Technology, PO Box 5196, Limbe, Malawi

**Keywords:** decision-making under uncertainty, water-energy-food nexus, Malawi, climate uncertainty, socio-economic development

## Abstract

Malawi depends on Lake Malawi outflows into the Shire River for its water, energy and food (WEF) security. We explore future WEF security risks under the combined impacts of climate change and ambitious development pathways for water use expansion. We drive a bespoke water resources model developed with stakeholder inputs, with 29 bias-corrected climate model projections, alongside stakeholder elicited development pathways, and examine impacts on stakeholder-elicited WEF sector performance metrics. Using scenario analysis, we stress-test the system, explore uncertainties, assess trade-offs between satisfying WEF metrics, and explore whether planned regulation of outflows could help satisfy metrics. While uncertainty from potential future rainfall change generates a wide range of outcomes (including no lake outflow and higher frequency of major downstream floods), we find that potential irrigation expansion in the Lake Malawi catchments could enhance the risk of very low lake levels and risk to Shire River hydropower and irrigation infrastructure performance. Improved regulation of lake outflows through the upgraded barrage does offer some risk mitigation, but trade-offs emerge between lake level management and downstream WEF sector requirements. These results highlight the need to balance Malawi's socio-economic development ambitions across sectors and within a lake-river system, alongside enhanced climate resilience.

This article is part of the theme issue 'Developing resilient energy systems'.

## 1. Introduction

Water-energy-food (WEF) nexus thinking has resonated with academics and stakeholders alike, despite the varied analytical frameworks, approaches and methods employed [1]. The WEF nexus integrates three sectors crucial to multiple sustainable development goals (SDGs) [2]. Water availability and demand are central to the nexus because of their importance for domestic water supply and use, hydropower generation, agricultural requirements and/or irrigation, fisheries and for sustaining ecosystem services. Nexus sector linkages are strong and changing fast in southern Africa [3]. Here, current and projected major increases in water demand for hydropower and irrigation expansion underpin regional scale development plans and government and multi-lateral agency aspirations for socio-economic development [4,5]. Malawi's recent ‘Vision 2063’ national development plan relies on similar reasoning and assumptions to focus on agriculture production, energy infrastructure and urbanization as key elements underpinning long-term sustainable development [6].

Across Africa, mainstreaming climate resilient development into on-going planning and policy making is vitally important [7]. Mainstreaming can help identify risks emerging from the complex interactions of coupled human-environment systems, myriad non-climate stressors (e.g. population growth, urbanization, land degradation etc.) and climate stressors (variability and change). Narrow sectoral priorities or ‘silo’ approaches within the nexus sectors have exacerbated vulnerabilities [8], necessitating moves to more integrated, cross-sectoral planning [9].

Decision-making under uncertainty (DMUU) approaches have applicability in WEF nexus decision contexts [10] because they can be customized to suit different sectors, decision contexts, data availability, levels of stakeholder engagement and sources of uncertainty [11]. An emerging concern regarding development plans is the possibility that interventions could exacerbate the risk of failure to meet performance objectives due to climate change or potentially lead to maladaptation [12]. DMUU approaches use an assess-risk-of-policy principle to analyse policy options, plans, alternative infrastructure designs and management options against uncertainties. Engaging with sectoral/multi-sectoral stakeholders to make development plans more resilient to climate change-related risks using DMUU approaches is crucial; however, African examples of such approaches across the WEF nexus at a river basin scale are limited. To achieve practical impact requires working with stakeholders from the outset to define and update research and development aims, identify key plans and management options, and link to wider national development aspirations. When done effectively, engagement can promote a co-development process in the design and use of research tools (e.g. models), share and fine-tune results, and develop insights that can inform on-going policy making and development plans.

In this paper, we explore the interactions between development pathways and climate change uncertainty, for a least developed country (LDC) context in Africa; Malawi. We focus on the transboundary Lake Malawi Shire River Basin (LMSRB) that supports greater than 90% of Malawi's hydropower and irrigation, water supply for its growing population, and environmental flows for an important biodiverse wetland, the Elephant Marsh, before the Shire River joins the Zambezi River. We use a Water Evaluation And Planning (WEAP) model developed with inputs and insights from multi-sectoral stakeholders [13] to analyse cross-sectoral risks.

Specifically, this paper focuses on the following research questions:

(1) How might existing irrigation expansion plans, and potential future expansion and intensification of irrigation in the Lake Malawi catchment, affect downstream Shire River hydropower, irrigation and environmental flow requirements?
(2) To what extent can regulating lake outflows help manage risks to stakeholder-identified hydropower and irrigation infrastructure, and environmental flow requirements in the Shire River, under uncertain changes in climate and upstream water use?

In §2, we elaborate on the decision context from a WEF nexus perspective within the LMSRB. In §3, we detail the methods, including stakeholder engagement, modelling approach and scenario framework. Results are presented in §4 and §5 discusses the results followed by brief conclusions from the study.

## 2. Decision context

**Figure 1.**
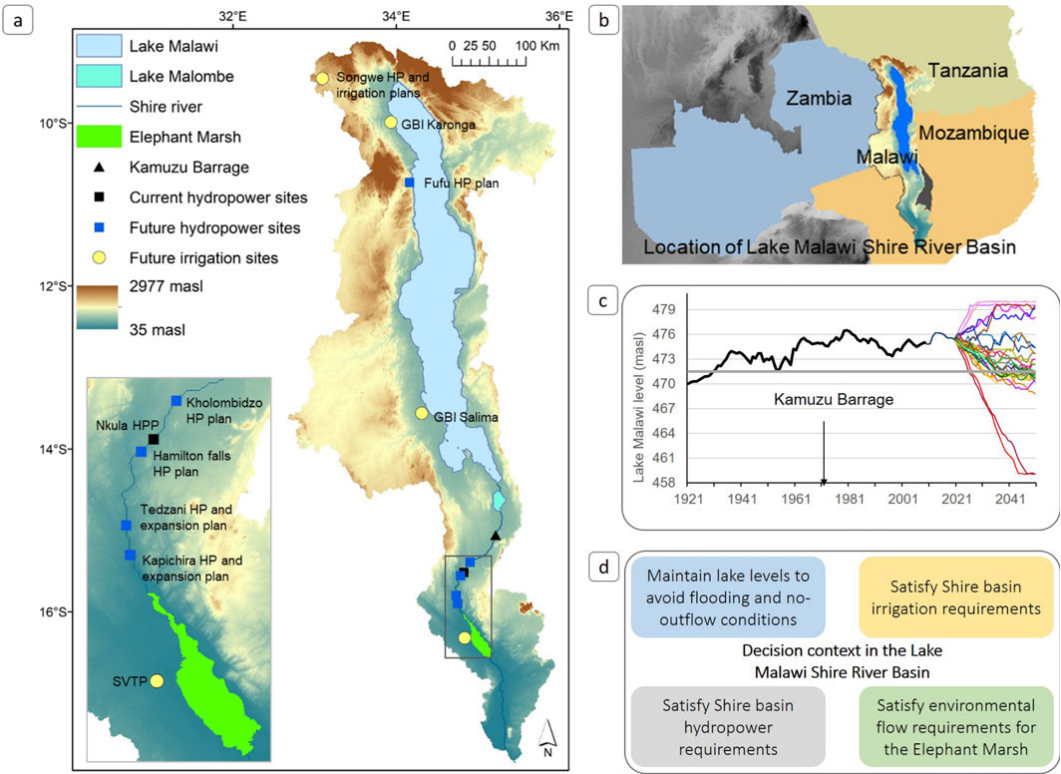
Decision context in the Lake Malawi Shire River Basin: (*a*) basin outline, elevation, key features and locations of the WEF sector plans, with more detail in the inset for Shire River hydropower and irrigation infrastructure and plans (SVTP, Shire Valley Transformation Project and HP, hydropower plant). (*b*) Location of the LMSRB, which covers most of Malawi and parts of Tanzania and Mozambique. (*c*) Observed Lake Malawi levels (black line), lake outflow threshold (471.5 masl), and potential future lake levels based on climate model projections (coloured lines) (cf. Bhave *et al*. [13]). (*d*) Four key objectives of the decision context from a WEF sector perspective. (Online version in colour.)

The LMSRB dominates Malawi's physical geography (figure 1*a*), has played a major role in the country's development to date, and is a crucial component of current and future plans for economic development. Malawi's development plans involve extensive irrigation and hydropower expansion ostensibly to address food insecurity and low access to electricity (approx. 9% of the population), among other objectives [6]. Infrastructure and large-scale agriculture-led development narratives are common in sub-Saharan Africa [14,15] and exert an important influence on investment patterns and national and multi-lateral priorities for development. While the origins, implementation and outcomes of such programmes are contested [16] recent increases in investment and infrastructure construction underscore the need for rigorous planning and recognition of the inter-dependencies that exist across scales and sectors and to explicitly evaluate climate resilience of such long-term investments (e.g. [17]). WEF sectors are highly exposed to climate variability (which is particularly high in the region, [18]), and given the long lifespan of infrastructure, its performance under potential future climate conditions needs to be considered.

Africa's third largest lake, Lake Malawi, overflows into the 410 km long Shire River, and together they form the LMSRB, the last major sub-basin of the Zambezi River Basin (figure 1*a*). Lake Malawi outflows sustain dry season Shire River flows, which since 1966 have been used for hydropower generation from the Nkula (1966), Tedzani (1973) and Kapichira (2000) hydropower plants (HPPs). Together their approximately 0.35 GW total installed capacity accounts for approximately 94% of electricity generation capacity of Malawi [13]. Currently, Malawi's largest irrigated area is a sugarcane growing region (approx. 16 500 ha) in the Lower Shire Basin, where a private enterprise and a farmers' cooperative extract water from the Shire River. Downstream of the irrigated area lies the 61 556 ha Elephant Marsh which supports human populations (small-scale fisheries and subsistence farming) and biodiversity (including endangered and vulnerable species, like the endemic fish species Sanjika (*Opsaridium microcephalum*)) [19].

Lake Malawi levels depend on runoff contribution from the mountainous regions around Lake Malawi that lie in Malawi, Tanzania and Mozambique, with lake inflow contributions of approximately 55%, approximately 41 and approximately 4%, respectively (figure 1*b*). The Kamuzu Barrage (built in 1965) enabled some regulation of outflows and management of lake levels; however, observations (starting in the early twentieth century), historical documents and paleoclimate evidence indicate that climate variability can cause lake levels to fall below the Lake Malawi Outflow Threshold (LMOT) of approximately 471.5 masl [20]. A multi-year dry period in the early 1990s and mid-2010s was associated with low lake levels and frequent disruption to electricity generation and outages throughout the country (figure 1*c*).

During wetter periods high lake levels pose flood risk to lakeshore and downstream Shire regions, such as those experienced in 1979–1980, when lake levels reached record highs. Extreme floods in the Lower Shire River due to high rainfall over the lower catchment (late 2015, 2018 and 2019) have also caused major socio-economic disruption [21]. High spatial and temporal variability of rainfall in the region, complex drivers of rainfall variability and limited ability of climate models to capture rainfall patterns in this region mean that there is deep uncertainty in future rainfall change [18]. Simulation of lake levels under 29 climate change projections (using historical barrage management rules) produces a wide range of lake level changes including extreme conditions with no lake outflow and higher frequency of major downstream floods [13] (figure 1*c*). The Kamuzu barrage was upgraded in 2018 to enable automated control of its release gates, using real-time rainfall and runoff data at key locations, primarily to optimize outflows for Shire River hydropower generation and irrigation requirements, but also for better management of lake levels. Before the upgrade, records of river flows and releases (and their underlying decisions) are very sparse, making it difficult to determine the precise influence of the barrage on fluctuations in lake level and outflows. However, it is possible to reconstruct lake levels confidently and Shire River outflows to a reasonable level [13]. Revised/new barrage operating rules are more transparent (§3) and will form the basis of how future developments are balanced against existing uses and climate variability.

Four key WEF sector priorities capture the complex decision-making context in the LMSRB (figure 1*d*). Decision makers have to balance potentially conflicting priorities between the interconnected Lake Malawi catchment and the Shire River Basin. Regulating lake levels within an acceptable range in a way that avoids no-outflow conditions and lakeshore flooding is important and this requirement has to be balanced against meeting Shire River Basin requirements for hydropower, irrigation and the environment.

## 3. Methods

### (a) Stakeholder engagement

The stakeholder engagement process comprised of stakeholder identification, initial personal interactions and sequential meetings with multi-sector stakeholders over a four-year period (2015–2019). Identification of participants was made through initial contacts within our project consortium. Other potential participants were identified by asking existing contacts to recommend a wide range of relevant individuals to engage with the study. We sought technical staff directly involved with water resources management, hydropower generation, agriculture, irrigation, domestic water supply, the environment and sectoral planners. Initial personal interactions helped introduce research objectives, and explain that the research approach involves both modelling/analysis and engagement with the aim of enhancing its value to stakeholders. We held three focused group discussions with representatives from the Departments of Irrigation, Surface Water and Agriculture Extension Services within the Ministry of Agriculture, Irrigation and Water Development (MoAIWD), the Department of National Parks and Wildlife, Electricity Supply Corporation of Malawi Ltd, the Shire River Basin Management Programme, and operators of the Kamuzu Barrage. These expert discussion forums aimed to facilitate a shared understanding of the system such that its main characteristics could be incorporated in the modelling, analytical framework and performance analysis. In the forums, stakeholders shared information and insights to help develop an initial conceptual water resources model, refine the resulting WEAP model, and discuss the analytical approach among private sector stakeholders, hydropower companies were engaged in the discussion forums, while we engaged the largest irrigation water user, Illovo Sugar Malawi, through personal visits. Stakeholders provided details regarding the infrastructure, including its operation, key performance needs and system vulnerabilities (cf. [13] for details on stakeholder inputs for WEAP model development). Performance needs and vulnerabilities help identify critical (for stakeholders of different sectors) features of the system where physical changes have notable socio-economic consequences. For example, long-term change in Shire River discharge would affect hydropower generation from the run-of-river hydropower plants making this a vulnerability, while the performance need is a minimum Shire River discharge to generate expected hydropower.

**Figure 2.**
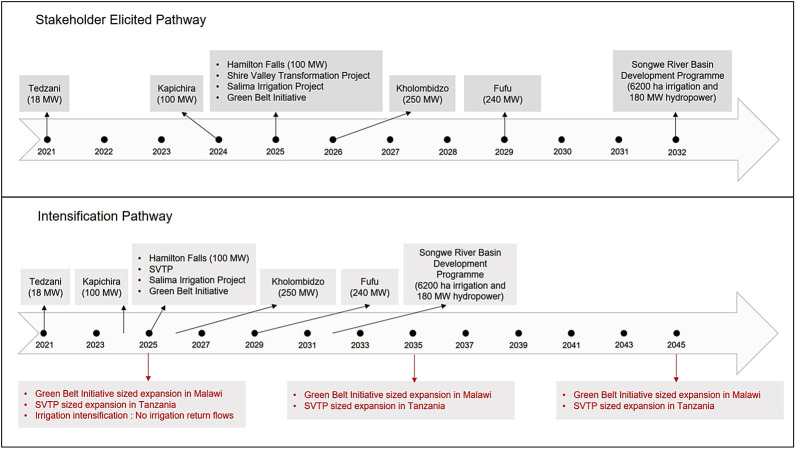
Development pathways: Top panel shows the timeline of the Stakeholder Elicited Pathway (SEP). Hydropower and irrigation infrastructure are planned for different years (also table 2) between 2021 and 2032. Bottom panel shows the timeline for the Intensification Pathway (IP) between 2021 and 2045. In the Intensification Pathway, the same infrastructure plans from SEP are shown above the timeline, and additional irrigation expansion and intensification in the Lake Malawi catchment (table 1 and Methods for more detail) is shown in red below the timeline. (Online version in colour.)

For this paper, we elicited stakeholder perceptions on uncertainties relating to drivers of future water availability and demand change, key characteristics and likelihood of hydropower and irrigation development plans in Malawi, and what were considered to be important performance metrics for WEF sectors and the environment in the Shire River Basin. We also asked about availability and details of water management options (table 1). Stakeholders emphasized the important role of the Kamuzu Barrage in managing outflows and identified the Kamuzu Barrage Operation Model (KABOM) (operationalized in 2019) as the main management option to meet downstream requirements. When asked about the drivers of water availability and demand in the basin, the stakeholders identified two main uncertainties; long-term change in rainfall and long-term change in water demand in Malawi and Tanzania. To address future rainfall uncertainty, we use 29 bias-corrected global climate model projections for Africa developed through the Future Climate for Africa programme [22]. These models are part of the Coupled Model Intercomparison Project (CMIP) 5, based on the RCP 8.5, accessed through the AMMA2050 project portal (http://amma2050.ipsl.upmc.fr/CMIP5_AFRICA/) and have been used in previous studies [13,18,24]. To address the uncertainty in water demand, we iteratively engaged stakeholders to elicit and construct two LMSRB development pathways focused on water demand from irrigation and hydropower. The first pathway is a stakeholder elicited pathway (SEP) based on current development plans identified by stakeholders as being realistic given business as usual. The second is an intensification pathway (IP) based on the SEP but with significant additional water demand in the Lake Malawi catchment designed to stress test the system (figure 2 and table 1, results §4.a).

**Table 1.** Components for scenario analysis identified with multi-sector stakeholders in the Lake Malawi Shire River Basin.

component	details and assumptions
climate change	period of analysis: 2021–2050
	29 bias-corrected Global Climate Model (GCM) projections at 0.5° × 0.5° spatial resolution [22]
	projections extracted for 35 LMSRB catchments using bilinear interpolation method, interpolated to a 0.05° spatial resolution
	catchment future reference evapotranspiration projections use variables derived from 29 GCM projections using the Penman–Monteith method (Lake Malawi evaporation not simulated). All variables for the Penman–Monteith method have also been bias-corrected based on method developed by Michelangeli *et al*. [23] (cf. [13]).
management option	Kamuzu Barrage Operation Model (KABOM) Release Strategy R1 for the Lake Malawi – Outflow Relationship at 0.5 m intervals for Lake Malawi levels (please see electronic supplementary material, figure 1 for further details)
performance metrics	Lake Malawi outflow threshold: lake levels are <471.5 masl
	lake shore flooding: lake levels are >477 masl
	Shire River hydropower: 265 cumecs discharge for peak power generation for each hydropower station
	Shire River irrigation: monthly requirement ranging from 37 to 50 cumecs
	environmental flow requirement for elephant marsh: 230 cumecs
	lower shire flood threshold: river discharge >1000 cumecs

### (b) The LMSRB model and performance metrics

In this study, we use a Water Evaluation and Planning (WEAP) model developed for the LMSRB by Bhave *et al*. [13]. This model is an improvement on previous efforts to simulate the complex lake-basin system because it incorporates the effects of the Kamuzu barrage regulation on Lake Malawi levels and downstream flows, and is computationally less intensive and practical for scenario analysis; all features specified as important during co-development with stakeholders. The model runs at a monthly time-step and is useful for exploring long-term changes in Lake Malawi levels and downstream flows, as well as simulating the impacts of Kamuzu Barrage operation changes. For this study, we modify the operation of the Kamuzu Barrage to reflect the recent upgrade and the characteristics of the KABOM model (table 1). The WEAP model does not incorporate future temperature increase to compute changes in lake evaporation because the lake response is highly uncertain. The current rainfall-evaporation balance is poorly understood due to sparse observations for on-lake rainfall and lake evaporation, future changes are uncertain because the effect of higher temperatures may be offset by other factors affecting evaporation (such as cloud cover), and land-atmosphere processes that generate feedback effects on the lake water balance are poorly understood (also see §5). Table 1 lists the key components elicited from stakeholders that inform the development pathway analysis and how they are incorporated into the WEAP model.

Six metrics are identified (using stakeholder prioritization) to represent the four main decision-relevant outcomes in terms of risk to infrastructure performance (table 1 and figure 1). The metrics and their derivation are as follows. A Lake Malawi metric targets a desirable range of lake levels (from 471.5 to 477 masl) with the aim of avoiding conditions of either no outflow or limited lakeshore flooding. The lower bound is a relatively hard threshold because outflow stops at 471.5 masl. Stakeholders identified the upper bound (477 masl) based on two criteria; the 1979–80 lake shore flooding and the design capacity of the upgraded Kamuzu Barrage. Different stakeholders identified different values, between 180 and 265 cumecs (cubic metres per second), for River Shire discharge necessary for peak hydropower generation at the existing stations. We select 265 cumecs because the stakeholders with most detailed knowledge (hydropower production and distribution companies) suggested this value. In 1979–1980, high lake levels and consequent high outflows contributed to lower Shire River discharge reaching the maximum observed value of approximately 1000 cumecs, which resulted in extensive flooding in the lower Shire (recent floods in 2015 and 2019 are more the result of high rainfall over the Shire River catchment, a process not included in our model). We therefore use this value as a performance metric for downstream flooding. For environmental flow requirements, no relevant previous study was available or provided by the stakeholders, based on which a metric could be developed. Given this, we use observed discharge at Chiromo gauging station, located at the end of the Elephant Marsh (figure 1). Since 1965 the Kamuzu Barrage has affected lower Shire River flows, and therefore environmental flow requirements need to be identified using pre-intervention discharge characteristics. Average monthly discharge at the Chiromo gauging station in the lower Shire, prior to the construction of the Kamuzu Barrage (for the period 1953–1965), indicates that the Q90 value (discharge which was equalled or exceeded for 90% of the time) is approximately 230 cumecs, which we use as an indicator of minimum environmental flows for the Elephant Marsh. For the Shire Valley Transformation Project (SVTP, the main irrigation water demand), we use the project's recommended monthly water supply requirements, which vary between 37 and 50 cumecs.

We use four scenario combinations as part of the scenario framework, which are described below.

(1) Baseline (hypothetical): This scenario uses observed climate for the period 1971–2000, historical water use and upgraded Kamuzu Barrage and operation rules. We choose this 30-year period because it includes incidences of high (1979–1980) and low lake levels (1995–2000), which helps capture the recent observed climatic variability. Although during the 1971–2000 period, the barrage and operation rules were actually different, we use the upgraded barrage and rules to allow direct comparison of impacts with simulations under future climate change and development pathways (under which the new barrage will be operational).
(2) Climate change with SEP: This scenario combination represents the period 2021–2050, with 29 climate change projections, SEP related water demand changes, and upgraded Kamuzu Barrage operation (§4.a).
(3) Climate change with IP: Represents the period 2021–2050, with 29 climate change projections, IP related water demand changes, and upgraded Kamuzu Barrage operation (§4.a).
(4) Climate Change with Old Barrage (hypothetical): This scenario for the period 2021–2050 uses climate change projections, historical water use and historical barrage operations. This combination is the same as presented in Bhave *et al*. [13] and is used here to show the effects of climate change assuming there has been no upgrade in the Kamuzu Barrage and its operation (i.e. the potential benefits/effects of the upgrade).

## 4. Results

### (a) Development pathways

The pathways represent two realizations of future changes in water demand associated with different assumptions about irrigation expansion. The SEP includes hydropower and irrigation plans (table 2 and figure 2), which are central to Malawi's planned socio-economic development in both the Lake Malawi and Shire River basin regions. Stakeholders identified a mix of ongoing projects (e.g. Tedzani and Kapichira hydropower sites and the SVTP irrigation plan), those still in the planning stage (e.g. Kholombidzo HP and the Green Belt Initiative for irrigation) including new (irrigation at Songwe, and hydropower at Fufu and Songwe sites in the north) and expansions (Tedzani, Mpatamanga and Kapichira hydropower plants in the south). The Shire Basin Development Programme (SRBDP), targeted for 2032, is based on a water sharing agreement between the riparian countries of Malawi and Tanzania, which includes significant new hydropower and irrigation. The plans comprising the SEP were identified under the assumption of business as usual conditions for planning and development.

**Table 2.** Stakeholder elicited hydropower and irrigation plans with details for the stakeholder elicited pathway (SEP), and details regarding the way plans are incorporated and expanded in the intensification pathway (IP).

infrastructure plans	details of planned infrastructure
Shire irrigation & hydropower plans (over the current 346 MW) and start year	Tedzani (18 Megawatts, MW) 2021
	Kapichira (100 MW) 2024
	Hamilton falls (100 MW) 2025
	Kholombidzo hydropower plant (250 MW) 2026
	Shire Valley Transformation Project (SVTP) (43 370 ha) 2025
stakeholder elicited pathway (SEP): irrigation plans in the lake catchment and start year	Green Belt Initiative (11 775 ha) 2025
	Salima Irrigation Project (7500 ha) 2025
	Songwe River Basin (6200 ha) 2032
intensification pathway (IP): lake catchment irrigation expansion (in addition to SEP plans) and start year	three phase irrigation expansion (2025–2035–2045) in Malawi and Tanzania.
	green belt initiative sized expansion in Malawi (a total of 35 325 ha in three phases)
	SVTP sized expansion in Tanzania (Ruhuhu basin) (a total of 130 110 ha in three phases)
	irrigation intensification: no irrigation return flows to the system, i.e. 100% consumption of water supplied for irrigation (contrary to an estimate of 15% return flow from all other irrigation areas)

The IP addresses stakeholder concerns about additional expansion of irrigation upstream in the Tanzanian part of Lake Malawi's catchment. It comprises upper estimates of irrigation expansion in Malawi and Tanzania, coupled with reduction of irrigation return flows, much greater than the stakeholders expected, as a stress-test of the system (table 2 and lower panel figure 2). Three phases of irrigation expansion are identified for Malawi and Tanzania (2025–2035–2045). In Tanzania, this comprises three additional irrigation projects, each the size of the SVTP. While such irrigation expansion scenarios could be considered towards the higher end, they are consistent with the Southern Agricultural Growth Corridor of Tanzania (SAGCOT) programme (see http://sagcot.co.tz/) investment blueprint. It envisages rapidly expanding irrigation in the Ludewa Cluster from approximately 8000 ha in 2020, to approximately 23 000 ha in 2025 and to approximately 37 000 ha by 2030 [25,26]. In Malawi, the IP implements three additional irrigation projects, each the size of the Green Belt Initiative. In all cases, we assume that the additional irrigation expansion has the same water demand per unit area as that estimated for ongoing/planned projects. In the SEP, the final development occurs with completion of the SRBDP in 2032, while the IP extends to 2045, when the third phase of irrigation expansion is envisaged to end. The different temporal evolution of the SEP and IP show the contrasting impacts of short-term and long-term changes in water demand in the Lake Malawi catchment regions.

### (b) Future risk profiles

**Figure 3.**
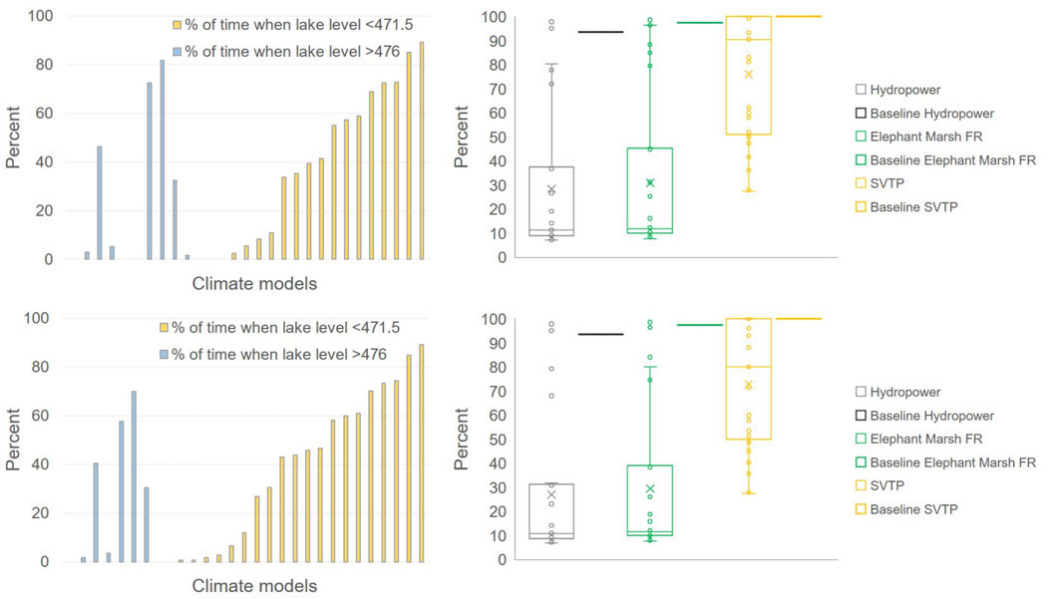
Future risk profiles: Upper panels present the decision metrics based risk profiles for Climate Change with SEP and lower panels for Climate Change with IP. Column graphs (left panels) show the per cent of time when lake levels are below the outflow threshold (471.5 masl) and above the threshold for lakeshore flooding (476 masl) for different climate model projections. Box-whisker plots (right panels) present the spread of projections for the reliability of water supply (per cent of time when requirements are met) for hydropower, environment (Elephant Marsh) and irrigation (SVTP). For the left panels (column graphs), climate models are sorted from low to high values for per cent of time when lake levels is less than 471.5 masl. (Online version in colour.)

We present results for five decision metrics with the scenario combinations ‘Climate Change with SEP’ and ‘Climate Change with IP’ compared with performance metrics under the Baseline scenario. Future risk profiles are derived from the suite of 29 GCM projections (figure 3). The wide range in results reflects the deep uncertainty in GCM projections and different sensitivities of the metrics. We focus on the range of potential future conditions as an indicator of the degree of uncertainty. Climate models do not always capture the climate processes and associated rainfall changes, particularly in regions with fewer observations [27]. Therefore, instead of focusing on specific projections and associated risks, we compare the range of projections between different scenarios to explore future risks. In the case of box-whisker plots, we find this analysis useful for understanding relative risks and trade-offs between different metrics. Risk is expressed as the percentage of time during the 30-year simulation period in which Lake Malawi levels go above/below the flood level/outflow threshold, and reliability of Shire River WEF sector performance metrics is expressed as the percentage of time (based on monthly values) during which the metrics are satisfied.

In the Baseline scenario, the lake level performance metrics are always met, i.e. the lake levels are never higher than 476 masl or lower than 471.5 masl. We find that the satisfaction of the Shire River performance metrics (box-whisker plots – right panels in figure 3) is high for the Baseline scenario; 93.7% for hydropower, 97.4% for the environment and 100% for irrigation in the SVTP. The Baseline risk (which assumes upgraded barrage rules) to the key decision metrics is therefore very low, while climate change projections indicate a wide range of potential future lake level changes and mostly a decline in reliability in Shire River metrics (figure 3). Projections for lake level metrics for the climate change with SEP scenario include four projections where lake levels are greater than 476 masl for greater than 25% of the time, and 12 projections where lake levels are less than 471.5 masl with different durations of time. This is a major increase in risk for crucial lake management metrics, due to combined effects of climate change and water demand increase. Enhanced agricultural water demand and agricultural intensification in the Climate Change with IP scenario reduces Lake Malawi inflows and results in slightly lower lake levels than for the Climate Change with SEP scenario (see electronic supplementary material, figure S3), and hence a marginally lower risk of lakeshore flooding. Conversely, the reduced lake inflows due to upstream water use lead to a substantial increase in the risk of going below the lake outflow threshold (13 projections indicating lake levels less than 471.5 masl for greater than 25% of the time).

Compared to the Baseline scenario, satisfaction of Shire River metrics is much lower for most projections under Climate Change with SEP and Climate Change with IP scenarios. Shire River metrics risk profiles for Climate Change with SEP and Climate Change with IP are quite similar (e.g. compare median values in figure 3, right panels). Even though the range of GCM projections shows deep uncertainty in rainfall change (wetter and drier) and Lake Malawi levels, the Shire River metrics almost exclusively show declines in reliability that in many cases are substantial. The reductions in reliability are much larger for the hydropower and environment metrics than the irrigation (SVTP) metric. Even for drier projections, water availability for the SVTP is only marginally affected by irrigation expansion and intensification in lake catchment regions. The main reason for this is that the absolute water demand for the irrigation metric (37–50 cumecs) is much lower than the hydropower and environment metrics (greater than 200 cumecs, table 1). Another reason is that the streamflow generated by rainfall upstream of the uptake for irrigation, especially during the rainy season, helps meet some of the irrigation requirements. This means that water supply for irrigation can be met even when supply for hydropower and environment is insufficient (cf. table 2).

Comparing the future risk profiles in figure 3, with the risk profile under the hypothetical scenario of Climate Change with Old Barrage (electronic supplementary material, figure S4), shows the effect of the upgraded barrage compared to the old barrage. The old barrage was operated in a way that ensured constant downstream flows to sustain Shire River hydropower, irrespective of the lake water levels. So, under the old barrage operation, potential future rainfall changes in the Lake Malawi catchment and the associated uncertainty are transferred to lake level changes, and the satisfaction of lake level metrics. While for some projections lake levels would rise, for many projections lake levels would fall below the outflow threshold for significant periods of time causing increases in both the risk of lakeshore flooding (six projections with lakeshore flooding for greater than 70% of the time) and no-outflow conditions (13 projections over 30% of the time). The upgraded barrage is better able to reduce the risk of lakeshore flooding (fewer projections with lake shore flooding) by allowing higher outflows when lake levels are higher, and allowing lower outflows when lake levels are lower, leading to a smoothing of lake level variability. However, these operation rules also affect the meeting of Shire River metrics (box plots in figure 3). For projections with lower rainfall outflows are constrained and lead to relatively lower satisfaction of Shire River metrics compared with the distribution of projections for the old barrage (box plots in electronic supplementary material, figure S4).

### (c) Risks to hydropower and Shire floods

In this section, we explore how potential future changes could affect Shire River hydropower generation and floods in the lower Shire River. For hydropower, we analyse hydropower generation as a percentage of the total installed capacity at that time, while for the Shire River floods we use the flooding metric (greater than 1000 cumecs; table 1 and §3.b). The two development pathways, SEP and IP, have the same sequence and timing of hydropower expansion plans on the Shire River, from the current 346 MW to 814 MW. Given that IP has more upstream water abstraction than SEP, assessing the difference in hydropower generation between the two scenarios could help better understand how the additional water demand could affect hydropower. High Shire River discharge can also damage the mechanical screens that protect the turbine (personal communication with representative of the electricity generation company in Malawi), besides contributing significantly to Shire River floods.

**Figure 4.**
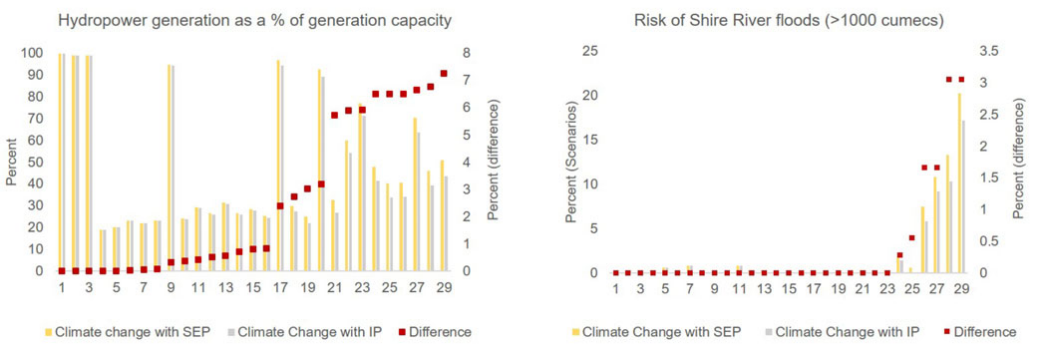
Risks for future hydropower generation and downstream Shire River floods. Left panel shows the future hydropower generation as a percentage of the generation capacity for Climate Change with SEP and Climate Change with IP (primary vertical axis). Red dots indicate the difference between the two scenarios (secondary vertical axis). The difference is calculated as Climate Change with SEP – Climate Change with IP. Right panel shows the risk of Shire River floods (>1000 cumecs, table 1 for details) under future scenarios. Difference (Climate Change with SEP—Climate Change with IP) is on the secondary vertical axis. For both panels, climate models are sorted from low to high difference between the two scenarios.

For the Baseline scenario, Shire River hydropower generation as a percentage of installed capacity is approximately 99%, suggesting that under historical climatic and water use conditions, the upgraded barrage operation would have helped maximize Shire River hydropower generation. For future scenarios Climate Change with SEP and Climate Change with IP (figure 4), we find that values differ significantly between the projections for different climate models (ranging from approx. 20 to approx. 100). However, there are relatively minor differences between the two development pathways, with a maximum difference between the two scenarios of approximately 8%. For projections with very high lake levels and very low lake levels upstream irrigation expansion has a marginal impact on hydropower generation, suggesting that when sufficient water is available (high lake levels) or outflow is restricted (low lake levels), there is limited additional impact of the upstream irrigation expansion. However, for projections with intermediate lake levels, the difference is larger. The slightly lower lake levels for the IP compared to the SEP at intermediate lake levels means greater constraints on lake outflows, and consequently, lower hydropower generation. Temporal evolution of simulated hydropower generation and installed capacity (electronic supplementary material, figure S2) indicates that for projections with lake levels falling below the LMOT hydropower generation is very low, indicating the severe impacts of no outflows on Shire River hydropower.

For the Baseline scenario, floods in the lower Shire River with discharge greater than 1000 cumecs (flooding metric) are not observed, suggesting that the improved barrage operation rules are useful in reducing the risk of downstream floods under historical climatic and water use conditions. For future scenarios (figure 4, right panel) we find that for four projections with the highest rainfall the Lower Shire River could be flooded for between 5 and 20 per cent of the time, which is a significantly higher risk compared to the Baseline scenario. For 23 climate model projections, there is a much lower risk of lower Shire River floods exceeding the flooding threshold. Also, for these projections comparing the Climate Change with SEP and Climate Change with IP scenarios, we find almost no difference between the per cent of time steps when the flooding threshold is crossed. This indicates that upstream water demand increase does not significantly impact the risk of Shire River floods for a majority of projections. However, for projections of higher rainfall (and consequently higher lake levels) there is up to 3% difference between the scenarios. This suggests that upstream increase in water demand and consequent reduction in Lake Malawi inflows could help reduce the risk of Lower Shire River flooding under future conditions with higher rainfall. Per cent exceedance curves (flood duration curves) for the two future scenarios (electronic supplementary material, figure S5) indicate that there is almost no perceptible difference between the profiles of the two future scenarios. This suggests that uncertainty in future lower Shire River discharge and its temporal characteristics is driven more by the future climate uncertainty than the uncertainty in future water demand change in the Lake Malawi catchment.

## 5. Discussion and conclusion

This study explores the different dimensions of WEF security risks under potential future changes in climate and water demand in a complex lake-basin system that is crucial for Malawi's economy and for its ability to address development challenges. In the modelling, we use a Water Evaluation and Planning (WEAP) model that has a number of assumptions owing to the complexity of the LMSRB and data sparsity. To improve the understanding of the system and support operational management and risk assessments in the basin, better and extensive monitoring of lake inflows and outflows and documentation of barrage management priorities is necessary. It is important to be clear about the assumptions and values inherent to any analysis of uncertainty, risk, water resources allocation priorities, and performance metrics. A key uncertainty here is the historical and potential future changes in lake rainfall-evaporation balance [13]. Better monitoring and access to data regarding rainfall-runoff processes in Lake Malawi catchments, on-lake rainfall, and lake evaporation are important to understand the lake water balance, and inform operational management of the Kamuzu Barrage. Climate process feedbacks between lake evaporation and basin rainfall are also crucial to understand potential future changes, and corresponding implications for water availability in the LMSRB. Our selection of performance indicators, while prioritized by stakeholders, is constrained by data availability and practical concerns about making the analysis manageable and communicable to a range of stakeholders. To enhance the relevance of such research for operational planning and decision-making, a larger suite of performance indicators with more detail in the risk characterization, and comprehensive sensitivity analysis would be necessary.

The government targets used here for irrigation and infrastructure expansion (SEP and IP) are towards the higher end of current targets, given the rate of development in the past and experience elsewhere in sub-Saharan Africa [28]. As such, they reflect maximal outcomes in terms of meeting government targets and have been used here to stress-test the system. More consultation could elicit wider views on alternative scenarios of basin development and barrage management, and their relative merits for different stakeholders and the environment. Further consideration of project design assumptions about efficiencies and returns are also warranted in view of past performance [29–31]. Finally, we present future risk without considering adaptation beyond the recent upgrade of the Kamuzu barrage. Through adaptive management and well-designed operating rules and improved irrigation practices, many of the risks identified here could potentially be mitigated further.

We show that a recent upgrading of the Kamuzu barrage and its operation rules has benefits, particularly in reducing lakeshore flooding risk. Under baseline climate conditions, the barrage satisfies LMSRB performance metrics for most of the time. At the river basin level, our analysis highlights important interdependencies; upstream, downstream and cross-sector. We obtain a wide range of results for the future, reflecting the deep uncertainty in climate model projections and different sensitivities of the performance metrics. While in some projections lake levels rise, causing increases in the risk of lakeshore flooding, for many projections lake levels fall below the LMOT for significant periods of time, leading to no-outflow conditions. Analysing the implications of low lake levels on maintenance of the barrage and hydropower infrastructure was not within the scope of this study, but could be an important issue worth exploring in terms of contingency planning and infrastructure management. Downstream of the lake, there is a decline in the satisfaction of Shire River performance metrics compared to the baseline. Of particular concern are very dry climate projections under which lake levels fall below the LMOT, when the barrage is no longer useful. These conditions would lead to a failure of the system, severely limiting the ability to satisfy hydropower and environmental flow metrics. Enhanced agricultural water demand and agricultural intensification in the Climate Change with IP scenario reduces Lake Malawi inflows and results in slightly lower lake levels than for the Climate Change with SEP scenario, and hence a marginally lower risk of lakeshore flooding. The four climate model projections with the highest rainfall cause the Lower Shire River to be flooded for between 5 and 20 per cent of the time, which is much more than under baseline conditions. Extreme rainfall in southern Malawi can lead to Shire River flooding, such as seen in 2015 and 2019, and potential future co-occurrence of high lake levels and high rainfall in the Shire River Basin would lead to very severe flooding in the lower Shire.

The uncertainty created by having many different projections of rainfall can be a challenge to decision-making, yet, the full climate risk profile is important for advising policy makers and guiding further research [32]. The most serious risks occur with several outlier models, which, depending on stakeholder risk appetite, could be further scrutinized for their representation of regional climate drivers and teleconnections. However, one approach to try to narrow the range of model projections by assessing model skill in simulating African climate in the region and exclude the weakest performing models did not greatly reduce the inter-model uncertainty and the range of climate change impacts in the LMSRB [24]. Given this, and underlying questions about the ability of climate models to satisfactorily simulate rainfall behaviour in the region [33], management and planning need to consider DMUU and adaptive management principles [10]. Besides long-term changes in mean rainfall, it will also be important to factor in temporal variability, from intra-seasonal to multi-decadal time scales. Under such deep uncertainty, it might be prudent to select options that align with other considerations (such as cost effectiveness and desirability), perform acceptably across wetter and drier conditions, and design and implement contingency planning for individual and sequences of extreme years. Governance of such policy processes may require strengthened capacity for cross-sectoral management, particularly between agencies responsible for water resources, energy, agriculture and the environment.

The study highlights how a single infrastructure, the Kamuzu Barrage, is of critical importance for the welfare of a large region. While the model-based simulations take into account the changed barrage operation rules, human intervention in barrage operation (e.g. to increase hydropower generation) could significantly affect how different performance metrics are or are not satisfied. Contingency plans might consider modified and adaptive barrage operating procedures and water allocation under both drier and wetter conditions and development of multi-agency drought and flood management plans for single and multi-year events. This would involve sequences of decision points across years, with increasingly stringent restrictions on releases after successive years of drought. Such plans would require careful analysis of climate variability at timescales ranging from sub-seasonal to multi-decadal, to identify trigger points for decisions and explore trade-offs between sectors and stakeholders.

The cost of completing and maintaining hydropower dams can exceed their benefits and alternatives or complementary options, such as solar, thermal or wind power, which can be expanded incrementally, are rapidly becoming more cost-competitive. This calls for more thinking around the different energy sources that are available to address development aspirations articulated in Malawi's Vision 2063 document [6]. Developing countries, like Malawi, are not just locked-in to climate change, but are also often locked-in on development plans based on assumptions of climate stationarity [4,18] among other things. Given future climate uncertainty, development plans require careful evaluation so as to avoid potential lock-in, incorporate flexibility and avoid mal-adaptation. Evolving approaches like adaptive management and DMUU are a useful way forward in responding to future uncertainties and risks, besides evaluating adaptation options and residual risks after adaptation.

## Data Availability

These article has no additional information.
